# Supplementary material for: Quantifying antibody kinetics and RNA detection during early-phase SARS-CoV-2 infection by time since symptom onset
Source: eLife. 2020 Sep 7;9:e60122. doi: 10.7554/eLife.60122 (PMC7508557; doi:10.7554/eLife.60122)
Supplement: Figure 1—source data 1. — SD: standard deviation; ‘N too low’ indicates a sample size too small to compute a mean and SD. [file elife-60122-fig1-data1.docx]

| **Antibody** | **Assay** | **Mean** | **SD** |
| --- | --- | --- | --- |
| IgG | ELISA (any antigen) | 13.3 | 5.7 |
| IgG | ELISA-NP | 13.3 | 5.7 |
| IgG | ELISA-Spike | 10.5 | 4.1 |
| IgG | MCLIA | 12.0 | 5.5 |
| IgG | LFIA | 11.9 | 5.3 |
| IgG | IFA | 7.5 | 0.8 |
| IgM | ELISA (any antigen) | 12.1 | 5.7 |
| IgM | ELISA-NP | *N too low* | *N too low* |
| IgM | ELISA-Spike | 12.6 | 5.8 |
| IgM | MCLIA | 11.6 | 4.4 |
| IgM | LFIA | 9.1 | 2.8 |
| IgM | IFA | 8.5 | 0.5 |
| IgG | Mild/Moderate (ELISA-NP) | 12.9 | 5.0 |
| IgG | Severe/Critical (ELISA-NP) | 15.5 | 6.8 |
| IgM | Mild/Moderate (ELISA-Spike) | 12.3 | 5.8 |
| IgM | Severe/Critical (ELISA-Spike) | 13.2 | 5.0 |
