# Supplementary material for: Quantifying antibody kinetics and RNA detection during early-phase SARS-CoV-2 infection by time since symptom onset
Source: eLife. 2020 Sep 7;9:e60122. doi: 10.7554/eLife.60122 (PMC7508557; doi:10.7554/eLife.60122)
Supplement: Figure 2—source data 1. — N: sample size (including interpolated samples). [file elife-60122-fig2-data1.docx]

| **IgG** | | | | |
| --- | --- | --- | --- | --- |
| **Day after symptom onset** | **Percentage positive** | **N** | **Lower 95% CI** | **Upper 95% CI** |
| 0 | 13 | 207 | 8 | 18 |
| 1 | 19 | 181 | 13 | 25 |
| 2 | 19 | 220 | 14 | 24 |
| 3 | 18 | 228 | 14 | 24 |
| 4 | 50 | 143 | 42 | 59 |
| 5 | 23 | 323 | 18 | 28 |
| 6 | 22 | 379 | 18 | 27 |
| 7 | 24 | 336 | 19 | 29 |
| 8 | 27 | 430 | 23 | 32 |
| 9 | 36 | 467 | 32 | 41 |
| 10 | 38 | 364 | 33 | 43 |
| 11 | 53 | 413 | 48 | 58 |
| 12 | 67 | 413 | 62 | 72 |
| 13 | 70 | 368 | 65 | 74 |
| 14 | 70 | 346 | 65 | 74 |
| 15 | 79 | 448 | 75 | 82 |
| 16 | 82 | 311 | 78 | 86 |
| 17 | 84 | 335 | 79 | 87 |
| 18 | 89 | 410 | 86 | 92 |
| 19 | 88 | 314 | 84 | 92 |
| 20 | 93 | 274 | 89 | 95 |
| 21 | 97 | 111 | 92 | 99 |
| 22 | 94 | 160 | 89 | 97 |
| 23 | 93 | 100 | 86 | 97 |
| 24 | 100 | 47 | 92 | 1 |
| 26 | 96 | 361 | 93 | 1 |
| 29 | 99 | 225 | 96 | 1 |
| 32 | 100 | 19 | 82 | 1 |
| 35 | 99 | 161 | 97 | 1 |
| 38 | 100 | 7 | 59 | 1 |
| 41 | 100 | 148 | 98 | 1 |
| 47 | 100 | 1 | 3 | 1 |
| 50 | 100 | 1 | 3 | 1 |
| 53 | 100 | 2 | 16 | 1 |
| 56 | 100 | 3 | 29 | 1 |
| 59 | 100 | 3 | 29 | 1 |
| 62 | 100 | 1 | 3 | 1 |
