# Supplementary material for: Quantifying antibody kinetics and RNA detection during early-phase SARS-CoV-2 infection by time since symptom onset
Source: eLife. 2020 Sep 7;9:e60122. doi: 10.7554/eLife.60122 (PMC7508557; doi:10.7554/eLife.60122)
Supplement: Figure 2—source data 2. — N: sample size (including interpolated samples). [file elife-60122-fig2-data2.docx]

| **IgM** | | | | |
| --- | --- | --- | --- | --- |
| **Day after symptom onset** | **Percentage positive** | **N** | **Lower 95% CI** | **Upper 95% CI** |
| 0 | 8 | 207 | 5 | 13 |
| 1 | 12 | 173 | 8 | 18 |
| 2 | 14 | 212 | 10 | 20 |
| 3 | 12 | 224 | 8 | 18 |
| 4 | 15 | 312 | 12 | 20 |
| 5 | 14 | 299 | 10 | 19 |
| 6 | 16 | 357 | 12 | 20 |
| 7 | 22 | 352 | 18 | 27 |
| 8 | 30 | 391 | 26 | 35 |
| 9 | 38 | 430 | 33 | 43 |
| 10 | 42 | 344 | 36 | 47 |
| 11 | 59 | 422 | 54 | 64 |
| 12 | 66 | 411 | 61 | 71 |
| 13 | 71 | 405 | 66 | 75 |
| 14 | 75 | 321 | 70 | 80 |
| 15 | 77 | 429 | 73 | 81 |
| 16 | 81 | 330 | 76 | 85 |
| 17 | 81 | 332 | 77 | 85 |
| 18 | 85 | 404 | 81 | 88 |
| 19 | 87 | 307 | 83 | 91 |
| 20 | 90 | 265 | 86 | 93 |
| 21 | 92 | 251 | 88 | 95 |
| 22 | 97 | 262 | 94 | 98 |
| 23 | 91 | 67 | 82 | 97 |
| 24 | 96 | 45 | 85 | 99 |
| 25 | 94 | 329 | 90 | 96 |
| 26 | 93 | 56 | 83 | 98 |
| 27 | 89 | 19 | 67 | 99 |
| 28 | 85 | 20 | 62 | 97 |
| 29 | 100 | 148 | 98 | 1 |
| 30 | 71 | 7 | 29 | 96 |
| 47 | 100 | 1 | 3 | 1 |
| 50 | 0 | 1 | 0 | 98 |
| 53 | 50 | 2 | 1 | 99 |
| 56 | 67 | 3 | 9 | 99 |
| 59 | 33 | 3 | 1 | 91 |
| 62 | 100 | 1 | 3 | 1 |
