# Supplementary material for: Quantifying antibody kinetics and RNA detection during early-phase SARS-CoV-2 infection by time since symptom onset
Source: eLife. 2020 Sep 7;9:e60122. doi: 10.7554/eLife.60122 (PMC7508557; doi:10.7554/eLife.60122)
Supplement: Figure 2—source data 3. — N: sample size (including interpolated samples). [file elife-60122-fig2-data3.docx]

| **RNA upper respiratory tract** | | | | |
| --- | --- | --- | --- | --- |
| **Day after symptom onset** | **Percentage positive** | **N** | **Lower 95% CI** | **Upper 95% CI** |
| 0 | 100 | 12 | 74 | 100 |
| 1 | 98 | 106 | 93 | 100 |
| 2 | 97 | 182 | 94 | 99 |
| 3 | 95 | 300 | 92 | 97 |
| 4 | 89 | 484 | 86 | 92 |
| 5 | 95 | 398 | 93 | 97 |
| 6 | 90 | 433 | 86 | 92 |
| 7 | 87 | 432 | 84 | 90 |
| 8 | 85 | 448 | 82 | 89 |
| 9 | 76 | 460 | 72 | 80 |
| 10 | 72 | 413 | 68 | 76 |
| 11 | 58 | 543 | 54 | 62 |
| 12 | 55 | 383 | 49 | 60 |
| 13 | 57 | 370 | 52 | 62 |
| 14 | 55 | 324 | 50 | 61 |
| 15 | 49 | 292 | 43 | 55 |
| 16 | 49 | 268 | 42 | 55 |
| 17 | 44 | 220 | 37 | 51 |
| 18 | 33 | 198 | 26 | 40 |
| 19 | 29 | 190 | 23 | 36 |
| 20 | 30 | 145 | 22 | 38 |
| 21 | 24 | 132 | 17 | 32 |
| 22 | 17 | 109 | 10 | 25 |
| 23 | 16 | 96 | 9 | 24 |
| 24 | 12 | 67 | 5 | 22 |
| 26 | 15 | 222 | 10 | 20 |
| 29 | 0 | 63 | 0 | 6 |
| 32 | 8 | 39 | 2 | 21 |
| 35 | 0 | 40 | 0 | 9 |
| 38 | 0 | 32 | 0 | 11 |
| 41 | 0 | 22 | 0 | 15 |
| 44 | 0 | 18 | 0 | 19 |
| 47 | 0 | 12 | 0 | 26 |
| 50 | 0 | 2 | 0 | 84 |
