# Supplementary material for: Quantifying antibody kinetics and RNA detection during early-phase SARS-CoV-2 infection by time since symptom onset
Source: eLife. 2020 Sep 7;9:e60122. doi: 10.7554/eLife.60122 (PMC7508557; doi:10.7554/eLife.60122)
Supplement: Figure 2—source data 4. — N: sample size (including interpolated samples). [file elife-60122-fig2-data4.docx]

| **RNA lower respiratory tract** | | | | |
| --- | --- | --- | --- | --- |
| **Day after symptom onset** | **Percentage positive** | **N** | **Lower 95% CI** | **Upper 95% CI** |
| 2 | 100 | 13 | 75 | 1 |
| 3 | 100 | 50 | 93 | 1 |
| 4 | 100 | 161 | 98 | 1 |
| 5 | 100 | 123 | 97 | 1 |
| 6 | 100 | 134 | 97 | 1 |
| 7 | 100 | 127 | 97 | 1 |
| 8 | 100 | 125 | 97 | 1 |
| 9 | 95 | 114 | 89 | 98 |
| 10 | 92 | 107 | 85 | 96 |
| 11 | 93 | 170 | 88 | 96 |
| 12 | 85 | 88 | 76 | 92 |
| 13 | 96 | 81 | 90 | 99 |
| 14 | 89 | 72 | 79 | 95 |
| 15 | 91 | 64 | 81 | 96 |
| 16 | 92 | 59 | 81 | 97 |
| 17 | 71 | 52 | 57 | 93 |
| 18 | 91 | 47 | 80 | 98 |
| 19 | 95 | 40 | 83 | 99 |
| 20 | 9 | 31 | 74 | 98 |
| 21 | 70 | 23 | 47 | 87 |
| 22 | 78 | 77 | 67 | 87 |
| 23 | 100 | 6 | 54 | 1 |
| 24 | 0 | 9 | 0 | 34 |
| 26 | 61 | 18 | 36 | 83 |
| 29 | 0 | 2 | 0 | 84 |
