# Supplementary material for: Quantifying antibody kinetics and RNA detection during early-phase SARS-CoV-2 infection by time since symptom onset
Source: eLife. 2020 Sep 7;9:e60122. doi: 10.7554/eLife.60122 (PMC7508557; doi:10.7554/eLife.60122)
Supplement: Figure 2—source data 5. — N: sample size (including interpolated samples). [file elife-60122-fig2-data5.docx]

| **RNA faeces** | | | | |
| --- | --- | --- | --- | --- |
| **Day after symptom onset** | **Percentage positive** | **N** | **Lower 95% CI** | **Upper 95% CI** |
| 0 | 100 | 4 | 40 | 1 |
| 3 | 100 | 6 | 54 | 1 |
| 4 | 85 | 13 | 55 | 98 |
| 5 | 88 | 16 | 62 | 98 |
| 6 | 91 | 66 | 81 | 97 |
| 7 | 75 | 81 | 64 | 84 |
| 8 | 91 | 87 | 83 | 96 |
| 9 | 87 | 82 | 77 | 93 |
| 10 | 88 | 73 | 78 | 94 |
| 11 | 90 | 77 | 81 | 95 |
| 12 | 88 | 74 | 78 | 94 |
| 13 | 89 | 73 | 80 | 95 |
| 14 | 90 | 62 | 80 | 96 |
| 15 | 87 | 52 | 74 | 94 |
| 16 | 80 | 51 | 67 | 90 |
| 17 | 80 | 44 | 65 | 90 |
| 18 | 77 | 47 | 62 | 88 |
| 19 | 61 | 41 | 45 | 76 |
| 20 | 43 | 35 | 26 | 61 |
| 21 | 37 | 30 | 20 | 56 |
| 22 | 27 | 22 | 11 | 50 |
| 23 | 39 | 28 | 22 | 59 |
| 24 | 14 | 14 | 2 | 43 |
| 26 | 16 | 43 | 7 | 31 |
| 29 | 12 | 26 | 2 | 30 |
| 32 | 0 | 10 | 0 | 31 |
| 35 | 0 | 6 | 0 | 46 |
| 38 | 0 | 6 | 0 | 46 |
| 41 | 0 | 6 | 0 | 46 |
| 44 | 0 | 6 | 0 | 46 |
| 47 | 0 | 2 | 0 | 84 |
