# Supplementary material for: Quantifying antibody kinetics and RNA detection during early-phase SARS-CoV-2 infection by time since symptom onset
Source: eLife. 2020 Sep 7;9:e60122. doi: 10.7554/eLife.60122 (PMC7508557; doi:10.7554/eLife.60122)
Supplement: Figure 3—source data 1. [file elife-60122-fig3-data1.docx]

| **Peak antibody level time** | | | |
| --- | --- | --- | --- |
| **Antibody/assay** | **Mean (day)** | **Lower 95% CrI** | **Upper 95% CrI** |
| IgM ELISA Spike | 19.1 | 15.6 | 22.4 |
| IgM ELISA NP | 12.2 | 7.8 | 16.2 |
| IgM MCLIA | 16.6 | 13.7 | 20.1 |
| IgG ELISA Spike | 20.4 | 16.8 | 24.1 |
| IgG ELISA NP | 15.2 | 12.8 | 17.2 |
| IgG MCLIA | 14.6 | 11.5 | 18.3 |
| IgG/IgM ELISA Spike | 20.0 | 17.6 | 22.4 |
| IgG/IgM ELISA NP | 14.3 | 12.0 | 16.1 |
