# Supplementary material for: Quantifying antibody kinetics and RNA detection during early-phase SARS-CoV-2 infection by time since symptom onset
Source: eLife. 2020 Sep 7;9:e60122. doi: 10.7554/eLife.60122 (PMC7508557; doi:10.7554/eLife.60122)
Supplement: Figure 3—source data 2. — Posterior differences between means were calculated by subtracting the posterior mean value for the antibody/assay in the second column from that of the first column, for each MCMC iteration. Differences were considered significant when zero was not included in the 95% credible interval (indicated in bold font). [file elife-60122-fig3-data2.docx]

| **Peak antibody level time posterior difference of means** | | | | |
| --- | --- | --- | --- | --- |
| **Antibody/assay** | **Antibody/assay** | **Difference (days)** | **Lower 95% CrI** | **Upper 95% CrI** |
| IgM MCLIA | IgG MCLIA | 2.0 | -2.6 | 7.0 |
| IgM MCLIA | IgG ELISA Spike | -3.8 | -8.5 | 1.3 |
| IgM MCLIA | IgM ELISA Spike | -2.5 | -7.2 | 2.2 |
| IgM MCLIA | IgG ELISA NP | 1.4 | -2.5 | 5.4 |
| IgM MCLIA | IgM ELISA NP | 4.4 | -1.1 | 9.8 |
| IgG MCLIA | IgG ELISA Spike | **-5.7** | **-10.4** | **-0.7** |
| IgG MCLIA | IgM ELISA Spike | -4.5 | -9.4 | 0.4 |
| IgG MCLIA | IgG ELISA NP | -0.6 | -4.9 | 3.3 |
| IgG MCLIA | IgM ELISA NP | 2.4 | -2.8 | 8.2 |
| IgM ELISA Spike | IgG ELISA Spike | -1.3 | -6.4 | 3.5 |
| IgM ELISA Spike | IgG ELISA NP | 3.8 | -0.2 | 8.0 |
| IgM ELISA Spike | IgM ELISA NP | **6.9** | **1.5** | **12.5** |
| IgG ELISA Spike | IgG ELISA NP | **5.1** | **0.8** | **9.2** |
| IgG ELISA Spike | IgM ELISA NP | **8.1** | **2.8** | **13.9** |
| IgG ELISA NP | IgM ELISA NP | 3.0 | -1.8 | 7.8 |
| IgG/IgM ELISA NP | IgG/IgM ELISA Spike | **-5.8** | **-9.2** | **-2.7** |
