# Supplementary material for: Quantifying antibody kinetics and RNA detection during early-phase SARS-CoV-2 infection by time since symptom onset
Source: eLife. 2020 Sep 7;9:e60122. doi: 10.7554/eLife.60122 (PMC7508557; doi:10.7554/eLife.60122)
Supplement: Figure 3—source data 3. [file elife-60122-fig3-data3.docx]

| **Growth rate** | | | |
| --- | --- | --- | --- |
| **Antibody/assay** | **Mean (1/day)** | **Lower 95% CrI** | **Upper 95% CrI** |
| IgM ELISA Spike | 0.41 | 0.34 | 0.49 |
| IgM ELISA NP | 0.68 | 0.42 | 1.03 |
| IgM MCLIA | 0.48 | 0.38 | 0.58 |
| IgG ELISA Spike | 0.39 | 0.32 | 0.46 |
| IgG ELISA NP | 0.53 | 0.45 | 0.61 |
| IgG MCLIA | 0.54 | 0.42 | 0.67 |
| IgG/IgM ELISA Spike | 0.39 | 0.34 | 0.44 |
| IgG/IgM ELISA NP | 0.55 | 0.48 | 0.64 |
