# Supplementary material for: Quantifying antibody kinetics and RNA detection during early-phase SARS-CoV-2 infection by time since symptom onset
Source: eLife. 2020 Sep 7;9:e60122. doi: 10.7554/eLife.60122 (PMC7508557; doi:10.7554/eLife.60122)
Supplement: Figure 3—source data 4. — Posterior differences between means were calculated by subtracting the posterior mean value for the antibody/assay in the second column from that of the first column, for each MCMC iteration. Differences were considered significant when zero was not included in the 95% credible interval (indicated in bold font). [file elife-60122-fig3-data4.docx]

| **Growth rate posterior difference of means** | | | | |
| --- | --- | --- | --- | --- |
| **Antibody/assay** | **Antibody/assay** | **Difference (1/day)** | **Lower 95% CrI** | **Upper 95% CrI** |
| IgM MCLIA | IgG MCLIA | -0.07 | -0.23 | 0.09 |
| IgM MCLIA | IgG ELISA Spike | 0.09 | -0.03 | 0.21 |
| IgM MCLIA | IgM ELISA Spike | 0.07 | -0.06 | 0.19 |
| IgM MCLIA | IgG ELISA NP | -0.05 | -0.18 | 0.08 |
| IgM MCLIA | IgM ELISA NP | -0.21 | -0.55 | 0.09 |
| IgG MCLIA | IgG ELISA Spike | **0.16** | **0.02** | **0.30** |
| IgG MCLIA | IgM ELISA Spike | 0.13 | -0.01 | 0.28 |
| IgG MCLIA | IgG ELISA NP | 0.02 | -0.14 | 0.18 |
| IgG MCLIA | IgM ELISA NP | -0.14 | -0.50 | 0.18 |
| IgM ELISA Spike | IgG ELISA Spike | 0.02 | -0.08 | 0.13 |
| IgM ELISA Spike | IgG ELISA NP | **-0.12** | **-0.23** | **-0.01** |
| IgM ELISA Spike | IgM ELISA NP | -0.27 | -0.62 | 0.01 |
| IgG ELISA Spike | IgG ELISA NP | **-0.14** | **-0.25** | **-0.03** |
| IgG ELISA Spike | IgM ELISA NP | **-0.30** | **-0.65** | **-0.02** |
| IgG ELISA NP | IgM ELISA NP | -0.16 | -0.50 | 0.14 |
| IgG/IgM ELISA NP | IgG/IgM ELISA Spike | 0.17 | -0.07 | 0.26 |
