# Supplementary material for: Quantifying antibody kinetics and RNA detection during early-phase SARS-CoV-2 infection by time since symptom onset
Source: eLife. 2020 Sep 7;9:e60122. doi: 10.7554/eLife.60122 (PMC7508557; doi:10.7554/eLife.60122)
Supplement: Figure 5—source data 1. — Note that sample sizes indicate those reported in the articles, not all of which were used for quantitative analysis. [file elife-60122-fig5-data1.docx]

| Authors | Year | Journal | doi | Antibody assay | IgG | IgM | NT | RNA | Sample size | Disease severity reported | Country |
| --- | --- | --- | --- | --- | --- | --- | --- | --- | --- | --- | --- |
| Adams et al. | 2020 | Wellcome Open Res | ﻿10.12688/wellcomeopenres.15927.1 | ELISA-Spike | Yes | Yes | - | - | 40 | Yes | United Kingdom |
| Du et al. | 2020 | J Med Virol | ﻿10.1002/jmv.25820 | - | Yes | Yes | - | - | 60 | No | China |
| Haveri et al. | 2020 | Euro Surveill | ﻿10.2807/1560-7917.ES.2020.25.11.2000266 | IFA | Yes | Yes | Yes | Yes | 1 | Yes | Finland |
| Lee et al. | 2020 | J Microbiol | 10.1016/j.jmii.2020.03.003 | - | - | - | - | Yes | 1 | Yes | Taiwan |
| Liu et al. | 2020 | medRxiv | ﻿10.1101/2020.03.18.20038018 | ELISA-NP | Yes | Yes | - | Yes | 63 | Yes | China |
| Liu et al. | 2020 | J Clin Microbiol | ﻿10.1128/JCM.00461-20 | ELISA-NP, ELISA Spike | Yes | Yes | - | - | 214 | No | China |
| Lou et al. | 2020 | Eur Respir J | ﻿10.1183/13993003.00763-2020 | ELISA-NP, ELISA Spike, MCLIA, Lat flow | Yes | Yes | - | Yes | 80 | No | China |
| Okba et al. | 2020 | Emerg Infect Dis | ﻿10.3201/eid2607.200841 | ELISA-NP, ELISA Spike | Yes | - | Yes | - | 3 | Yes | France |
| Thevarajan et al. | 2020 | Nature Medicine | ﻿10.1038/s41591-020-0819-2 | IFA | Yes | Yes | - | Yes | 1 | Yes | Australia |
| To et al. | 2020 | Lancet Infect Dis | ﻿10.1016/S1473-3099(20)30196-1 | ELISA-NP, ELISA Spike | Yes | Yes | Yes | Yes | 23 | Yes | China |
| Wölfel et al. | 2020 | Nature | ﻿10.1038/s41586-020-2196-x | IFA | Yes | Yes | Yes | Yes | 9 | Yes | Germany |
| Xiang et al. | 2020 | Clin Infect Dis | ﻿10.1093/cid/ciaa461 | ELISA-NP | Yes | Yes | - | - | 85 | No | China |
| Xiao et al. | 2020 | J Infect | ﻿10.1016/j.jinf.2020.03.012 | MCLIA | Yes | Yes | - | - | 34 | No | China |
| Yongchen et al. | 2020 | Emerg Microbes Infect | ﻿10.1080/22221751.2020.1756699 | Lat flow | Yes | Yes | - | Yes | 21 | Yes | China |
| Young et al. | 2020 | JAMA | ﻿10.1001/jama.2020.3204 | - | - | - | - | Yes | 18 | No | China |
| Zhang et al. | 2020 | medRxiv | ﻿10.1101/2020.03.28.20043059 | - | - | - | - | Yes | 23 | Yes | China |
| Zhang et al. | 2020 | Aging | ﻿10.18632/aging.103102 | ELISA-NP | Yes | Yes | - | - | 6 | No | China |
| Zhang et al. | 2020 | Emerg Microbes Infect | ﻿10.1080/22221751.2020.1729071 | ELISA-NP | Yes | Yes | - | Yes | 39 | No | China |
| Zhao et al. | 2020 | Clin Infect Dis | ﻿10.1093/cid/ciaa344 | ELISA-NP, ELISA Spike | Yes | Yes | - | Yes | 173 | Yes | China |
| Zhou et al. | 2020 | Nature | ﻿10.1038/s41586-020-2012-7 | ELISA-NP, ELISA Spike | Yes | Yes | Yes | Yes | 7 | Yes | China |
| Zou et al. | 2020 | N Engl | ﻿10.1056/NEJMc2001737 | - | - | - | - | Yes | 18 | Yes | China |
